# Pathogenic Differences between Nipah Virus Bangladesh and Malaysia Strains in Primates: Implications for Antibody Therapy

**DOI:** 10.1038/srep30916

**Published:** 2016-08-03

**Authors:** Chad E. Mire, Benjamin A. Satterfield, Joan B. Geisbert, Krystle N. Agans, Viktoriya Borisevich, Lianying Yan, Yee-Peng Chan, Robert W. Cross, Karla A. Fenton, Christopher C. Broder, Thomas W. Geisbert

**Affiliations:** 1 Galveston National Laboratory, University of Texas Medical Branch, Galveston, TX, USA; 2 Department of Microbiology and Immunology, University of Texas Medical Branch, Galveston, TX, USA; 3 Department of Microbiology and Immunology, Uniformed Services University of the Health Sciences, Bethesda, Maryland, USA

## Abstract

Nipah virus (NiV) is a paramyxovirus that causes severe disease in humans and animals. There are two distinct strains of NiV, Malaysia (NiV_M_) and Bangladesh (NiV_B_). Differences in transmission patterns and mortality rates suggest that NiV_B_ may be more pathogenic than NiV_M_. To investigate pathogenic differences between strains, 4 African green monkeys (AGM) were exposed to NiV_M_ and 4 AGMs were exposed to NiV_B_. While NiV_B_ was uniformly lethal, only 50% of NiV_M_-infected animals succumbed to infection. Histopathology of lungs and spleens from NiV_B_-infected AGMs was significantly more severe than NiV_M_-infected animals. Importantly, a second study utilizing 11 AGMs showed that the therapeutic window for human monoclonal antibody m102.4, previously shown to rescue AGMs from NiV_M_ infection, was much shorter in NiV_B_-infected AGMs. Together, these data show that NiV_B_ is more pathogenic in AGMs under identical experimental conditions and suggests that postexposure treatments may need to be NiV strain specific for optimal efficacy.

Some 15 years ago, Nipah virus (NiV) emerged and was shown to be a previously unknown paramyxovirus, now classified along with Hendra virus and Cedar virus within the *Henipavirus* genus. NiV causes febrile encephalitis^1^ and severe respiratory disease^2^ in humans with a fatality rate as high as 100% in some outbreaks^3^. Pteropid fruit bats have been identified as the reservoir for NiV in nature^4^,^5^,^6^ although pigs served as an amplifying host during the first outbreak of NiV in Malaysia^7^. Additionally, there are numerous other mammalian species that are susceptible to NiV infection^5^,^8^,^9^,^10^,^11^,^12^,^13^.

Genetic analysis has identified at least two strains of NiV responsible for outbreaks in different geographical areas^14^. The Malaysia strain (NiV_M_) caused the initial outbreak of NiV from 1998–99 in Malaysia and Singapore in which over 270 people were infected with about 40% case fatality rate (CFR)^7^,^14^ with an additional 2014 outbreak in the Philippines with a CFR of ~52%, although the strain identification is based off a short read of the genome^15^ so it is not completely certain which strain of the NiV caused this outbreak. The Bangladesh strain (NiV_B_) however has caused repeated outbreaks, varying in number, in Bangladesh and northeast India with outbreaks occurring almost every year between 2001–2015^3^,^16^,^17^,^18^,^19^. The outbreaks of NiV_B_ have had higher CFRs averaging about 75%^17^ with human-to-human transmission also observed^20^,^21^. The observations that these two strains reportedly display differences in CFRs and human-to-human transmission are interesting as there is 91.8% nucleotide homology between the genomes^14^.

Clinical data from NiV outbreaks has revealed several key differences between patients infected with NiV_M_ and NiV_B_. First, NiV_B_ has a shorter average incubation period and a more narrow range for the incubation period than NiV_M_^2^,^22^,^23^. Second, most cases of NiV_B_ included respiratory symptoms while few patients infected with NiV_M_ presented with respiratory symptoms^1^,^2^,^19^. Third, few cases in the Bangladeshi and Indian NiV_B_ outbreaks reported myoclonus^24^, while a significant proportion of patients from the Malaysian outbreak presented with segmental myoclonus^1^,^22^,^24^ as well as the fatal cases in the Philippines presenting with an acute encephalitis syndrome^15^. Fourth, the source of the virus in the Bangladeshi and Indian outbreaks is either unknown in some cases or has been traced to consumption of contaminated fruit or date palm sap, followed by human-to-human transmission and nosocomial spread^20^,^21^,^23^,^25^,^26^,^27^, whereas the source of the virus in the Malaysian outbreak is known to be from pigs, which served as an amplifying host^28^. Unlike examples found in NiV_B_ outbreaks^21^, there were only two reported cases of potential transmission from human-to-human in the Malaysian outbreak, neither of which presented with symptoms during the outbreak^29^,^30^, although there were some reported cases of documented human-to-human transmission in the Philippines outbreak^15^. Fifth, there is an increased rate of vomiting with NiV_B_ infection compared with the NiV_M_^1^,^2^,^24^.

Despite the fact that there have been only one or two outbreaks of NiV_M_ while repeated outbreaks of NiV_B_ have occurred, almost all pathogenesis and vaccine research has utilized NiV_M_ rather than the potentially more medically relevant NiV_B_ strain. Recently, the important questions surrounding these two strains of NiV have been investigated, with particular interest in attempts to compare the strains in animal models that, to date, have accurately reflected the NiV_M_ disease syndromes seen in humans. There are numerous animal models used to study NiV_M_ (reviewed in ref. 31) with the hamster^11^,^32^, ferret^12^,^33^, and African green monkey (AGM)^13^,^34^ models most faithfully recapitulating NiV human disease. To date, studies comparing NiV_M_ and NiV_B_ in hamsters^35^ and in ferrets^36^ have been performed with conflicting results.

While the hamster and ferret models faithfully reproduce the neural and respiratory disease pathology seen in human cases of NiV, recent comparison of NiV_M_ and NiV_B_ in these model systems did not reflect the apparent differences between the strains as observed in humans^35^,^36^. However, the observations of strain difference in hamsters^35^, although opposite of the human cases, and the slight difference in oral shedding between the strains in the ferret model^36^, suggested that differences seen in human cases could be due to the strain of virus. To advance an understanding of this issue, we examined the pathogenesis of NiV_M_ and NiV_B_ in the AGM model using virus stocks at the same passage, identical virus dose, with all animals challenged at the same time and by exactly the same route. Here, we report that infection of AGMs with NiV_M_ and NiV_B_ under identical experimental conditions led to similar observations as seen in the human cases between the strains with NiV_B_ being more pathogenic for respiratory tissues, suggesting that one of the main differences in human outbreaks could be attributed to the strain of NiV. Additionally, we showed that the therapeutic window for NiV_B_ treatment of AGMs with the human monoclonal antibody m102.4 is shorter than for NiV_M._

## Results

### Deep sequencing of NiV_M_ and NiV_B_ isolates

To compare the P2 NiVstocks used in this study to the published NiV nucleotide sequences, the viral stocks were deep sequenced. These data are presented in Supplementary Table 1. There were 10 differences of sufficient frequency to note between the P2 stock of NiV_M_ and the reference sequence GenBank Assession number AJ627196.1. Of these, two were non-coding, seven were silent mutations, and one led to a single amino acid change in the N protein. There were four mutations of sufficient frequency to note between the P2 stock of NiV_B_ and the reference sequence GenBank Assession number AY988601.1. Of these, one was non-coding, and the other three led to single amino acid changes: one in the M protein and two in the F protein. In this present study, the NiV strains were passage matched at P2 however, we were interested in a previous passage 3 NiV_M_ used in an antibody therapeutic study^37^. Deep sequencing was also used to analyze the NiV_M_ P3 stock. Compared to the reference sequence GenBank Assession number AJ627196.1, there are two mutations at sufficient frequency to note. One of these was silent and one led to a single amino acid change in the Glycoprotein (G).

### NiV_M_ and NiV_B_ growth kinetics in target endothelial cells

**Figure 1:**
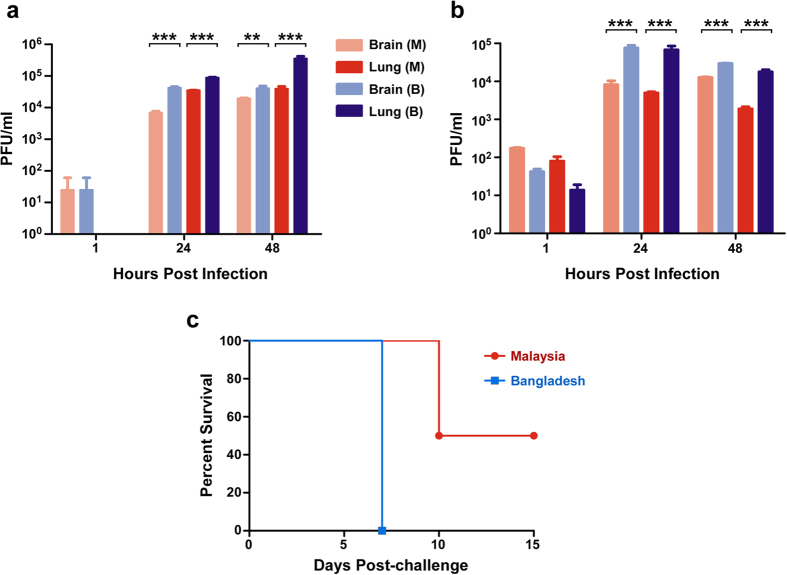
NiV_M_ and NiV_B_ comparison in human endothelial cells and AGMs. Growth kinetics in endothelial cell lines at MOI 5 (**a**) and primary endothelial cell cultures at MOI 1 (**b**). Lighter colors represent brain endothelial cell experiments and darker colors represent lung endothelial cell experiments; Red hues: NiV_M_; Blue hues: NiV_B_. Error bars represent standard deviation of the mean. ANOVA with Dunnett’s Multiple Comparison Test; n = 4. **p-value < 0.01; ***p-value < 0.001. Limit of detection 25 PFU/ml. (**c**) Kaplan-Meier survival curves for AGMs infected with NiV_M_ (red) and NiV_B_ (blue). Log-rank (Mantel-Cox) Test; n = 4 for both cohorts. *p-value < 0.05.

To assess the growth kinetics of NiV_M_ and NiV_B_ stocks, which were passaged in the same cell lines and same number of times, we infected brain (hCMEC/D3) or pulmonary endothelial cell lines (hpmecst1.6r) or primary brain (HBCMEC) or pulmonary (HPMEC) cells as these mimic the assumed *in vivo* target cells. In the endothelial cell lines NiV_M_ titers seemed to peak by 24 hpi whereas NiV_B_ produced higher levels of infectious virus at 48 hpi in the pulmonary cell line (Fig. 1a, dark blue). This result was intriguing as NiV_B_ outbreaks have more respiratory sequelae associated with human cases when compared to NiV_M_. When we further characterized the two strains in the primary cell lines, NiV_B_ was found to grow to higher titers than NiV_M_ in both the brain (Fig. 1b, light blue) and pulmonary (Fig. 1b, dark blue) primary endothelial cells suggesting that NiV_B_ has better fitness in target endothelial cells. In addition, Vero E6 cells are used to propagate NiV isolates, and the peak titers observed at 48 hpi are comparable between NiV_B_ and NiV_M_. While previous studies in hamsters revealed that NiV_M_ had better fitness in BHK-21 cells and subsequently in hamsters^35^, these data in human endothelial cells led us to compare the two NiV strains in the AGM model of disease as it best recapitulates the human NiV disease course.

### NiV_M_ and NiV_B_ challenge and disease in AGMs

We previously described the development of a NiV_M_ disease model in AGMs which recapitulated clinical signs and pathology observed in NiV-mediated disease in humans^13^. Clinical signs in this model include severe depression, respiratory disease leading to acute respiratory distress, neurologic disease, reduced activity, and a time to death ranging from 9 to 12 days. To date NiV_B_ challenge of AGMs has yet to be described; additionally, a direct comparison of the Malaysia and Bangladesh strains of NiV in AGMs has not been examined. To explore the differences between these NiV strains we challenged two cohorts of four AGMs each by the i.n. and i.t. routes with either NiV_M_ or NiV_B_ and observed them over the course of 15 days post-challenge. After challenge, subjects from each cohort were observed and scored for clinical signs of disease based on depression, recumbency, respiratory quality, and neurological signs (Table 1). All four animals in the NiV_B_ cohort succumbed to NiV-mediated disease on day 7 (Fig. 1c, blue); whereas two of the four animals in the NiV_M_ cohort succumbed to NiV-mediated disease on day 10 and the two remaining animals survived until the end of the 15 day study (Fig. 1c, red) the results of which were significant by Log-rank (Mantel-Cox) test with a p-value < 0.05 (Fig. 1c, *). All animals from the NiV_M_ cohort reached low, initial clinical scores on day 7, due to respiration quality and depression, with all animals scoring over the course of 4–7 days and two of the four animals (O8000 and O8023) reaching clinical scores that required euthanasia on day 10 post-challenge (Fig. 1c, red). As found in Table 1, each animal in the NiV_M_ cohort experienced depression, fever, a loss of appetite, dyspnea, lymphopenia, thrombocytopenia, and hypoalbuminemia with noticeable nasal exudates from three of the four animals (O7912, O7935, and O8000) with epistaxis associated with O8000; additionally, two of the four animals experienced tremors (O7935 and O8000). The NiV_B_ cohort also initially scored on day 7; however, these animals displayed a more severe clinical score due to depression, respiration quality, and recumbency ultimately succumbing to NiV-mediated disease on day 7 (Fig. 1c, blue). Animals in the NiV_B_ cohort also experienced fever, depression, a loss of appetite (up to 3 days earlier than NiV_M_ cohort, O8021 Table 1), severe dyspnea, lymphopenia, thrombocytopenia, hypoalbuminemia, noticeable nasal exudate with epistaxis in O8022, and tremors noted for O8046. Overall, the clinical scores for each group were associated with the period of observed clinical illness where animals infected with NiV_M_ had a more protracted illness as seen in the clinical illness column in Table 1.

### NiV_M_ and NiV_B_ load in swabs, blood, and tissues

**Figure 2:**
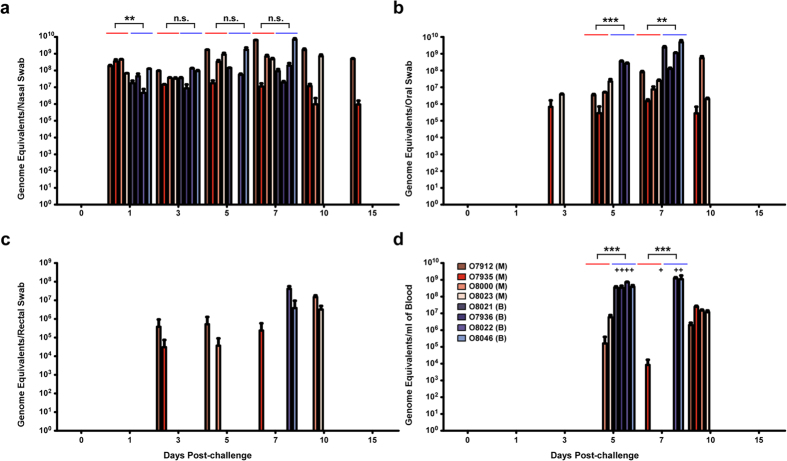
Viral load in swabs and blood. NiV_M_ (red hues) and NiV_B_ (blue hues) viral RNA genomic equivalents detected by qRT-PCR from sampling at days 0, 1, 3, 5, 7, 10, and 15 in nasal swabs (**a**), oral swabs (**b**), rectal swabs (**c**), or circulating in blood (**d**). Plus sign denotes infectious virus isolation from sample day. Error bars represent standard deviation. ANOVA with Dunnett’s Multiple Comparison Test was performed on the mean values of all animals from each group; n = 4. n.s., not significant; **p-value < 0.01; ***p-value < 0.001.

To determine if there was a difference in virus replication between the NiV_M_ - and NiV_B_ - infected cohorts, shedding of virus was assessed by qRT-PCR on nasal, oral, and rectal swabs (Fig. 2a–c, respectively) with viremia also screened by qRT-PCR on whole blood samples (Fig. 2d). High levels of NiV GEq were detectable in nasal swabs for both cohorts starting at day 1 and through day 7 with the NiV_M_ group having positive nasal swabs through day 15 (Fig. 2a). The NiV_M_ oral swabs had detectable NiV GEq starting at day 3 and continuing through day 10 while the NiV_B_ oral swabs were positive starting at day 5 and reaching up to two logs higher levels at day 7 (Fig. 2b). NiV GEq were detected at levels up to 3 logs higher in the blood of NiV_B_-infected animals at day 5 and 7 post-challenge when compared to the NiV_M_ cohort. Infectious virus was isolated from all whole blood NiV_B_ RNA positive samples (Fig. 2d,+) while one NiV_M_ whole blood sample was positive from O8023 on day 7 (Fig. 2d,+). Typically, detection of infectious virus from whole blood samples that are NiV RNA positive has been inconsistent in the NiV_M_ model^13^; however, the higher levels of circulating NiV_B_ in blood may have contributed to the consistent isolation of infectious virus from RNA positive samples.

**Figure 3:**
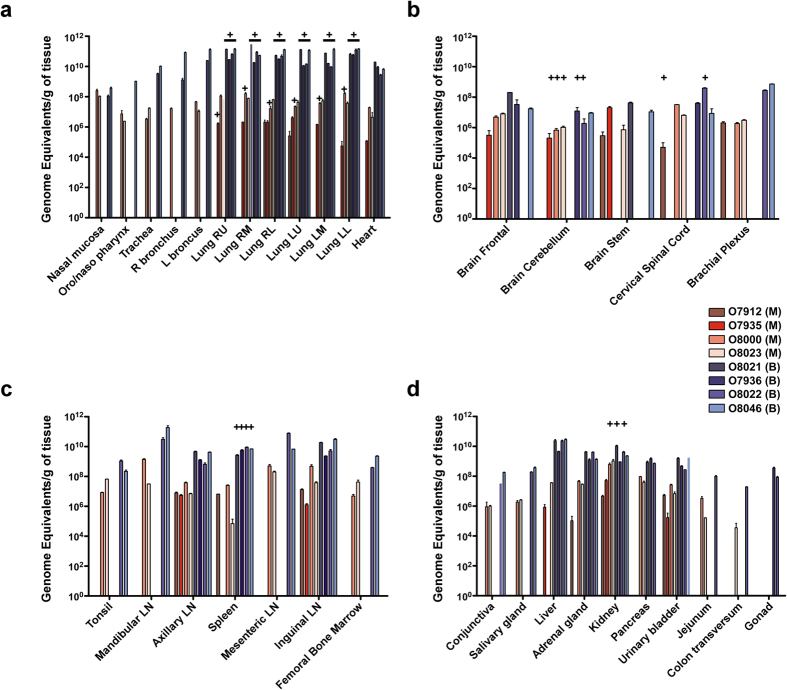
Viral load in tissues. NiV_M_ (red hues) and NiV_B_ (blue hues) viral RNA genomic equivalents detected by qRT-PCR from tissues at study endpoint for each AGM in pulmonary associated tissues (**a**), neural tissues (**b**), lymphoid tissues (**c**), or other typical NiV-infected tissues (**d**). Plus sign denotes infectious virus isolation from sample; underlined plus sign denotes virus isolated from every AGM in group for that particular sample. R (right), L (left), RU (right upper), RM (right middle), RL (right lower), LU (left upper), LM (left middle), LL (left lower), LN (lymph node). Error bars represent standard deviation.

To further examine the replication of the viruses in AGMs, we performed qRT-PCR on tissue samples collected after animals succumbed to NiV-mediated disease or at the end of the 15 day study (O7912 and O7935). Strikingly, the detectable levels of NiV_B_ GEq in the respiratory tissues were 3 logs higher from the bronchi to the lower lobes of the lungs when compared to the NiV_M_ group (Fig. 3a). However, NiV GEq detected in neural tissue were similar among all neural tissues sampled (Fig. 3b). The results from the tissues in AGMs were similar to growth of NiV_M_ and NiV_B_ in microvascular lung endothelial cell and microvascular brain endothelial cell lines where NiV_B_ grew to higher titers in the lung endothelial cells but the titers were equivalent in the brain endothelial cells (Fig. 1a) whereas NiV_B_ grew to higher titers in both primary microvascular lung and brain endothelial cells (Fig. 1b). Examination of the lymphoid tissues revealed higher levels of NiV_B_ GEq in the axillary and inguinal lymph nodes as well as in the spleen (Fig. 3c) while additional tissues with higher levels of NiV_B_ included the liver and adrenal gland (Fig. 3d).

To determine if the isolation of infectious virus from tissues would reflect the difference observed between the groups in regard to the whole blood samples, tissues were selected for virus isolation from the qRT-PCR positive tissues from at least one tissue from each panel (Fig. 3). Infectious NiV_B_ was isolated from all lung lobes for each animal (Fig. 3a, ±) whereas one animal per lobe had infectious NiV_M_ isolated including O7912 from the right upper lobe (Fig. 3a, +). Three animals (O7935, O8000, and O8023) from the NiV_M_ cohort had infectious virus isolated from the cerebellum (Fig. 3b, +); two animals from the NiV_B_ group were positive for infectious virus in the cerebellum as well (O7936 and O8022). Cervical spinal cord samples from one animal in each cohort had infectious virus isolated; O7912 from the NiV_M_ group and O8022 from the NiV_B_ group. Only animals from the NiV_B_ group had infectious virus isolated from spleen samples (Fig. 3c, +) while one animal from the NiV_M_ group (O8000) and two animals from the NiV_B_ (O8021 and O8022) had virus isolated from the kidney (Fig. 3d, +).

### Gross pathology, histopathology, and immunohistochemical analysis of NiV-infected AGMs

**Figure 4:**
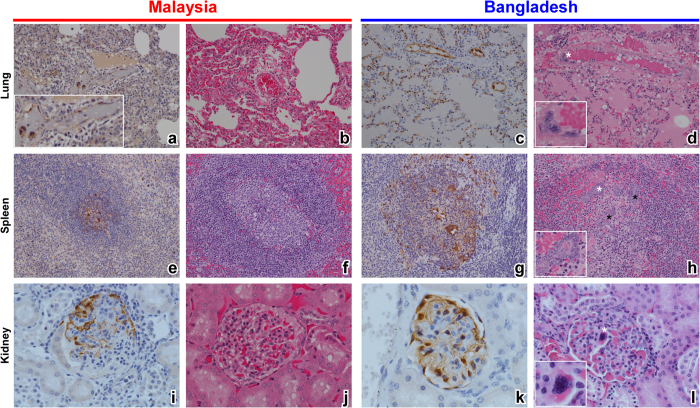
H&E and immunohistochemistry of AGM lung, spleen, and kidney. Representative H&E of lung (**b,d**), spleen (**f,h**), and kidney (**j,l**) and representative immunohistochemistry for NiV antigen of lung (**a,c**), spleen (**e,g**), and kidney (**i,k**). Cells immunopositive for NiV antigen appear brown. NiV_M_ in left panels and NiV_B_ in right panels. (**a**) inset a magnification of endothelial cells immunopositive for NiV antigen in lung. (**d**) inset a magnification of area marked by white asterisk showing an endothelial cell syncytium in lung. (**h**) inset a magnification of area marked by white asterisk showing large syncytium in the spleen; black asterisks mark other areas of syncytia in the spleen. Representative normal splenic germinal center architecture in (**f**) whereas the white pulp is disrupted in (**h**). (**l**) inset a magnification of area marked by white asterisk showing a syncytium of endothelial cells in a glomerular tuft. Lung and spleen tissue section images taken at 20X magnification and kidney tissue section images taken at 40X magnification.

**Figure 5:**
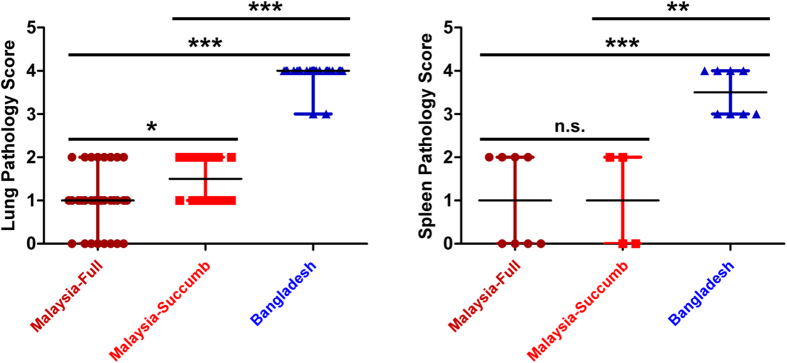
Histopathology scores for lung and spleen. (**a**) Lung scores based on two histopathology categories for all lung lobes and all indicated animals 1) perivascular edema and vasculitis and 2) pneumonia-interstitial, subacute with fibrin and edema. (**b**) Spleen scores based on histopathology associated with lymphoid necrosis, lymphoid necrosis, hemorrhage, and fibrin. Scoring index based on percent affected tissue: 0- no lesion (none); 1- minimal change (10% and less); 2- mild change (11–25%); 3- moderate change (26–50%); 4- marked change (51–75%); 5- severe change (76–100%). Malaysia-Full represents n = 4 NiV_M_ cohort. Malaysia-Succumb represents n = 2 of NiV_M_ cohort that succumbed to NiV_M_. Bars show distribution of scoring and median scores. A Mann-Whitney test comparing the sum rank of each group was used: (**a**) *p-value = 0.0111, ***p-value < 0.0001; (**b**) n.s. not significant, **p-value = 0.0041, ***p-value = 0.0004.

Typical gross lesions found in the urinary bladder and brain (Supplementary Fig. 1) of NiV-infected animals were similar between the NiV_M_ and NiV_B_ groups; however, examination of respiratory tissues revealed a characteristic failure of lung lobes to collapse with multifocal areas of hemorrhage and congestion (Supplementary Fig. 2a,b) with an excess of serosanguinous fluid in the pleural cavity (Supplementary Fig. 2c,d; asterisks) for both NiV_M_ and NiV_B_. However, overall the NiV_B_ lung lobes were more engorged with edema as the lobes were more rounded (Supplementary Fig. 2b, arrows) (i.e., inflated balloon) upon examination when compared to the NiV_M_ lung lobes (Supplementary Fig. 2a, arrowheads) (i.e., collapsed balloon). Histopathological and immunohistochemical examination revealed interstitial pneumonia, edema, hemorrhage, and vascular pathology, indicated by the endothelial syncytial cell formation (Fig. 4d, asterisk/inset), were more severe in the NiV_B_ cohort (Fig. 4a–d). Ordinal scoring of the lung lesions revealed a statistically significant difference between the NiV_B_ cohort (blue) and all NiV_M_ animals (dark red) as well as to the NiV_M_ cohort that succumbed (bright red) (Fig. 5a). The NiV_M_ cohort that succumbed also had a significant difference in lung lesions compared to the two animals that did not succumb, though not as significant as compared to NiV_B_ (Fig. 5a). Additionally, the overall quantity and distribution of NiV antigen was more prevalent in the NiV_B_ lung sections (Fig. 4a vs. c), with immunohistochemical ordinal scoring revealing an median of 0 for the NiV_M_ cohort and 4 for the NiV_B_ cohort. These observations suggest that the efficiency of oxygen exchange and blood flow in the lungs would be more reduced in the NiV_B_ animals compared to the NiV_M_ animals and indeed we observed an accentuation of the normal lobular pattern in the liver as indicated by pale multifocal to coalescing foci on the capsular surface of the liver (Supplementary Fig. 2d, inset). This observation coupled with such striking lung pathology is suggestive of reduced cardiovascular efficiency and passive hepatic congestion in which vulnerable zones of hepatocytes are subjected to periods of hypoxia and undergo vacuolar degeneration with adjacent hepatocyte zones having distended/congested sinusoids which is further suggestive of the apparent increase in lung pathology in the NiV_B_ tissues.

Histopathological and immunohistochemical examination of the spleen and kidney lesions revealed differences that were also striking between the NiV_M_ and NiV_B_ animals that succumbed to disease. The lymphoid follicular architecture of the splenic white pulp was minimally altered in the NiV_M_ animals (Fig. 4f), whereas partial to complete obliteration of the white pulp with hemorrhage, lymphoid depletion (Fig. 4h), and numerous, massive syncytial cells (Fig. 4h, asterisks/inset) were noted in the NiV_B_ group. Ordinal scoring of the spleen lesions revealed a statistically significant difference between the NiV_B_ cohort (blue) and all NiV_M_ animals (dark red) as well as to the NiV_M_ cohort that succumbed (bright red) (Fig. 5b). The NiV_M_ cohort that succumbed did not have a significant difference in spleen lesions compared to the two animals that did not succumb (Fig. 5b). Strong immunoreactivity for NiV antigen was also more abundant in the spleens of NiV_B_ animals (Fig. 4e vs. g), with immunohistochemical ordinal scoring revealing a median of 1 for the NiV_M_ cohort and 3 for the NiV_B_ cohort. In the kidney tissues the NiV_M_ and NiV_B_ had minimal interstitial lymphoplasmacytic infiltration, glomerular congestion, fibrin deposition, and immunolabeling for NiV antigen (Fig. 4i–l). However, the NiV_B_ kidney tissues overall had stronger immunolabeling for NiV antigen (Fig. 4i vs. k) and more endothelial cell syncytial formation in the glomerulus and medullary vessels (Fig. 4l, asterisk).

### Time of m102.4 treatment is crucial for protection of AGMs from NiV_B_-mediated disease

**Figure 6:**
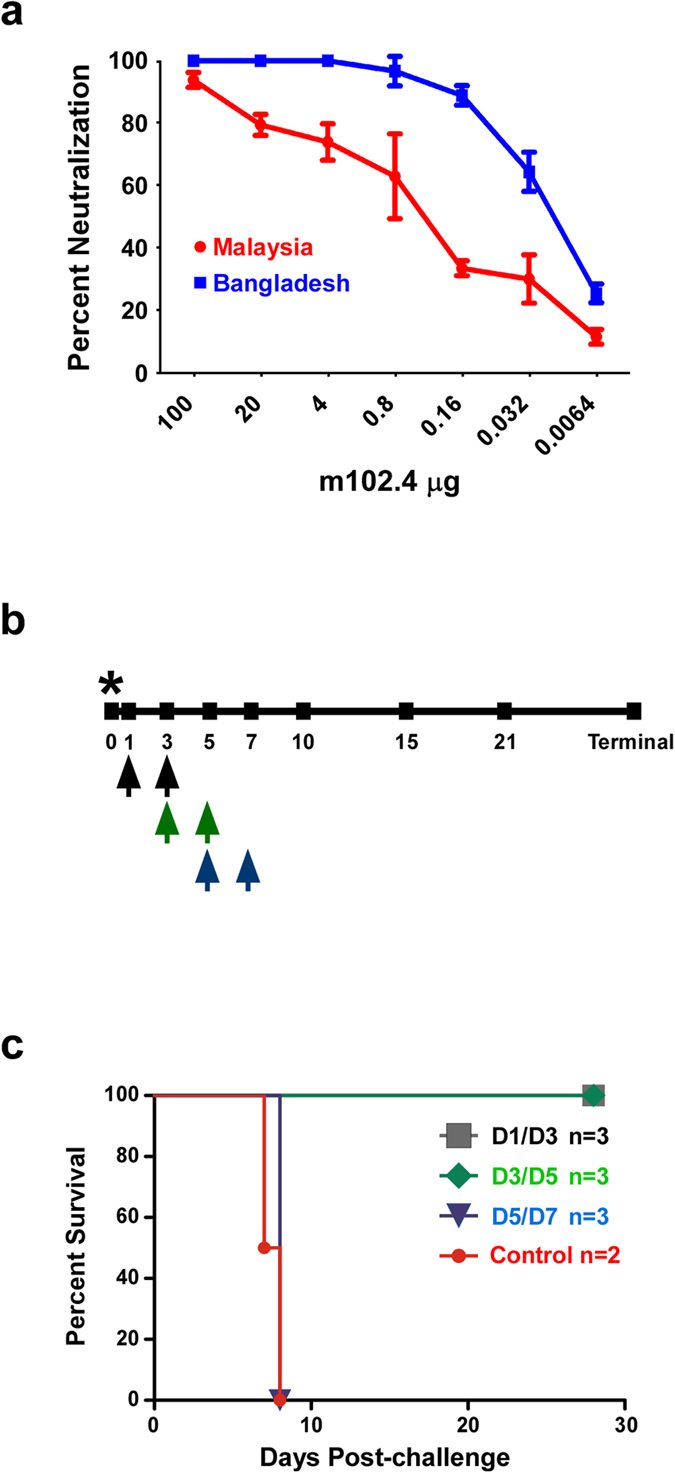
Human mAb m102.4 activity against NiV_B_ *in vitro* and *in vivo*. (**a**) Percent neutralizing antibody dose response of m102.4 against NiV_M_ (red) and NiV_B_ (blue) infection in Vero cells. Error bars show standard deviation. (**b**) Diagram of the m102.4 treatment regimen and sampling days post NiV_B_ challenge in AGMs. Black arrows; D1/D3 treatment. Green arrows; D3/D5 treatment. Blue arrows; D5/D7 treatment. * depicts the day of NiV_B_ challenge. (**c**) Kaplan-Meier survival curves for the D1/D3 group (gray, n = 3), D3/D5 group (green, n = 3), the D5/D7 group (blue, n = 3), and the control group (red, n = 2). Log-rank (Mantel-Cox) Test; *p-value < 0.05 comparing the NiV_B_ D5/D7 cohort with the previously reported data from the NiV_M_ D5/D7 cohort^37^.

Previously, we showed that administration of the NiV neutralizing human mAb m102.4 beginning as late as 5 days after NiV_M_ challenge of AGMs resulted in complete protection at a time when animals were viremic and showed signs of NiV disease^37^. Considering the higher rates of replication, increased lung pathology, and shorter time to death observed in AGMs challenged with NiV_B_ when compared to NiV_M_, we were interested in determining whether or not initiating a similar post-challenge treatment regimen with m102.4 could protect AGMs against NiV_B_. An *in vitro* assessment of the neutralizing capability of m102.4 against NiV_B_ revealed that m102.4 was at least as potent at neutralizing NiV_B_ when compared to NiV_M_ (Fig. 6a). With the encouraging m102.4 *in vitro* neutralization data, we proceeded to assess the protective efficacy of m102.4 against NiV_B_ challenge. The m102.4 antibody was administered to AGMs i.v. twice, with the first dose given 1 day after infection and the second dose 3 days after virus challenge (Fig. 6b, black arrows). To determine if the m102.4 therapeutic window could be extended, we treated two additional groups: one group received m102.4 beginning at 3 days after challenge and again at 5 days after challenge (Fig. 6b, green arrows), and the third treatment group received m102.4 at 5 days after infection and again at 7 days after virus challenge (Fig. 6b, blue arrows). No adverse reactions were observed following m102.4 infusion in any of the 9 treated animals. The two control subjects, consistent with the pathogenesis study above, succumbed to NiV_B_ disease on days 7 and 8 (Fig. 6c, red), respectively, and had signs of severe sustained behavior changes (depression, decreased activity), loss of appetite, fever (O7923), dyspnea, lymphopenia, thrombocytopenia, hypoalbuminemia, and changes in liver enzymes (Table 2). In contrast, subjects treated with m102.4 at day 1 after infection and again two days later (D1/D3) survived challenge (Fig. 6c, gray) and showed little to no clinical signs of disease with normal hematology and minor clinical chemistry results (Table 2). The three animals treated at day 3 after infection and again two days later (D3/D5) survived NiV_B_ challenge (Fig. 6c, green) and did not show any changes in behavior; these animals had mild changes in clinical pathology as compared to the D1/D3 treatment group (Table 2). The third treatment group differed as all three animals treated at day 5 after infection and again two days later (D5/D7) succumbed to NiV_B_-mediated disease on day 8 post-challenge (Fig. 6c, blue); all three also had signs of severe sustained behavior changes (depression, decreased activity), loss of appetite, fever, dyspnea, lymphopenia, thrombocytopenia, hypoalbuminemia, and changes in liver enzymes as seen in the control group (Table 2). The surviving animals from the D1/D3 and D3/D5 groups all had detectable neutralizing antibody after treatment and through the end of the study unlike the control group and much higher than the observed levels for the D5/D7 group (Supplementary Table 2). These data show that in the AGM NiV_B_ disease model, the window to therapeutically intervene is clearly shorter when compared to a previous NiV_M_ study in which all D5/D7 (Fig. 6c, historical) had a significant effect on survival when compared to the current D5/D7 group (Fig. 6c, *)^37^.

### m102.4 treatment effect on NiV_B_ load and subsequent humoral response in AGMs

**Figure 7:**
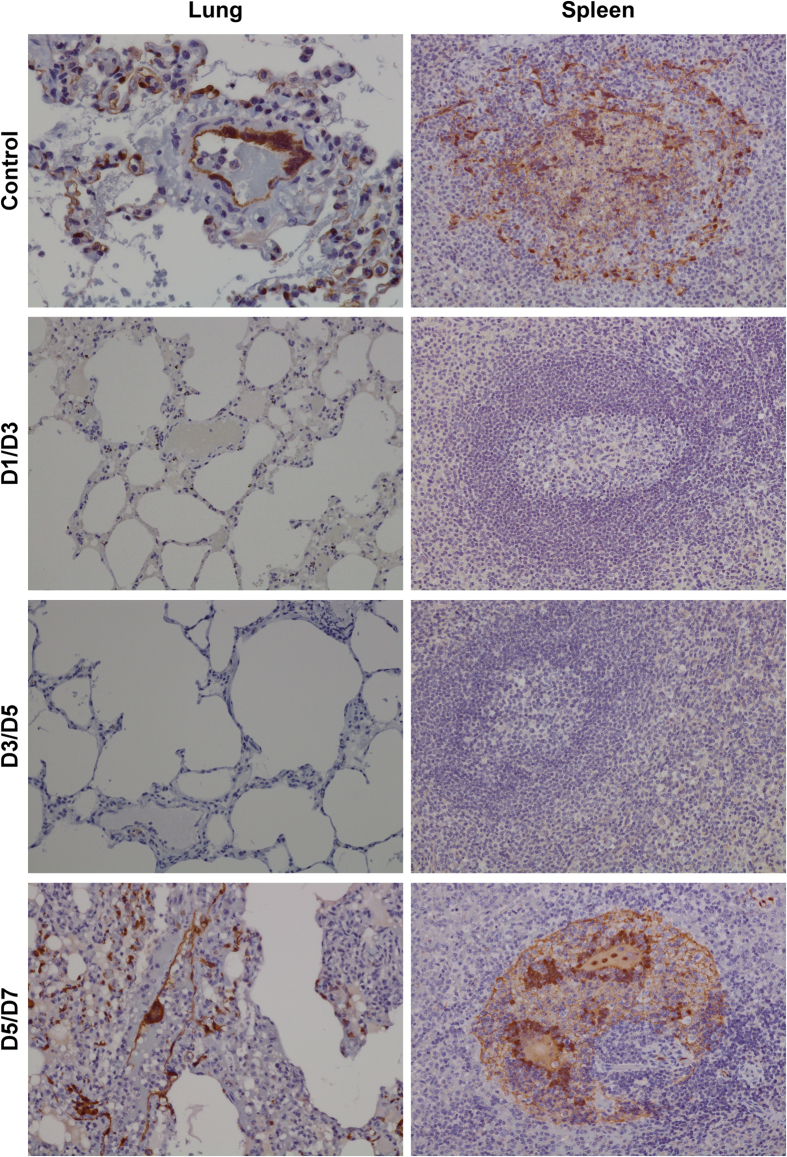
Immunohistochemistry of AGM tissue after NiV_B_ challenge and m102.4 treatment. Lack of NiV antigen in representative m102.4 D1/D3 and D3/D5 treated tissues and localization of NiV antigen in representative control and D5/D7 treated tissues by immunohistochemical staining. Lung and spleen were labeled with an N protein-specific polyclonal rabbit antibody and images taken at 20X magnification.

NiV_B_ replication was assessed by qRT-PCR on nasal, oral, and rectal swabs (Supplementary Fig. 3a,b and b, respectively) with viremia also screened by qRT-PCR on whole blood samples (Supplementary Fig. 3d). NiV_B_ genome equivalents were observed in nasal swabs in all groups starting on day 3 (Supplementary Fig. 3a) and varied between groups in oral swabs from the control (red), D3/D5 (green), and D5/D7 (blue) groups with the D1/D3 group (gray) only positive on day 3 (Supplementary Fig. 3b). Viral RNA was detected in blood samples at day 3 from the D1/D3 group (O8214), the D3/D5 group (O8149), and at high levels from day 5 for the D5/D7 (blue) and control (red) groups (Supplementary Fig. 3d). High levels of NiV_B_ RNA were also detected systemically in the tissues of all animals in the D5/D7 and control groups (Supplementary Fig. 3e). These data are consistent with the detection of viral antigen by immunohistochemistry where only the control and D5/D7 groups were positive for antigen in the major target organs lung and spleen (Fig. 7).

**Figure 8:**
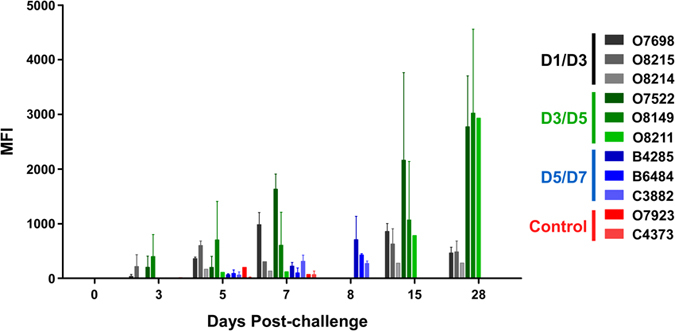
Seroconversion of AGMs post-challenge and m102.4 treatment. Detection of NiV F specific antibodies from m102.4 treated and non-treated AGMs. D1/D3 group (gray, n = 3), D3/D5 group (green, n = 3), the D5/D7 group (blue, n = 3), and the control group (red, n = 2). Mean fluorescence intensities (MFI) are shown on the y-axis and represent binding of specific Ig (IgG, and IgM) to NiV F. Error bars represent the standard deviation of fluorescence intensity across 100 beads for each sample.

Considering we detected circulating NiV_B_ RNA in all groups examined, we wanted to assess the role of the host immune response in mediating protection for the D1/D3 and D3/D5 groups from NiV_B_ disease. To do this, we measured circulating levels of antibodies directed against the NiV F glycoprotein in sera of the NiV-infected AGMs (Fig. 8). Anti-NiV F antibodies were present in sera of all D1/D3 and D3/D5 m102.4-treated AGMs with antibody levels increasing by day 28 (O7522, O8149, and O8211) while the D5/D7 group and two control AGMs did not seroconvert to high levels.

## Discussion

Two strains of NiV have been responsible for human outbreaks in different geographical regions^14^,^15^. Clinical data from these NiV outbreaks has revealed several key differences between patients infected with NiV_M_ and NiV_B_. Despite the fact that there have been only one or two outbreaks of NiV_M_ while repeated outbreaks of NiV_B_ have occurred, almost all pathogenesis, therapeutic, and vaccine research has utilized NiV_M_ models rather than the potentially more relevant NiV_B_ strain based on number of outbreaks and potential for human-to-human transmission. Recently, the important questions surrounding these two strains of NiV have been investigated, with particular interest in attempts to compare the strains in animal models that, to date, have accurately reflected the NiV_M_ disease syndrome seen in humans. Numerous animal models have been used to study NiV_M_ (reviewed in ref. 31) with the hamster^11^,^32^, ferret^12^,^33^, and AGM^13^,^34^ models most faithfully recapitulating NiV human disease. To date, studies comparing NiV_M_ and NiV_B_ in hamsters^35^ and in ferrets^36^ have been performed with conflicting results.

Comparison of NiV_M_ and NiV_B_ infection in hamsters revealed that NiV_M_ had more fitness in baby hamster kidney cells and caused a more rapid and pathogenic disease course *in vivo*^35^; however, respiratory tract lesions were found to be similar between the two strains in a separate study^38^. Interestingly, the higher fatality rate and more rapid disease progression of NiV_M_-infected hamsters compared to NiV_B_ differ from what has been reported in human cases. Additionally, the hamster model has recently revealed a NiV_B_ food-borne infection and transmission model replicating the assumed outbreak conditions in India and Bangladesh^39^. From the hamster model data, it has been suggested that there are no intrinsic differences between the two strains of NiV^38^.

To further compare NiV_M_ and NiV_B_, the ferret model was employed for pathogenic examination. After inoculation, it was found that the disease course, clinical signs, and pathology of the two strains were similar in ferrets^36^. However, a few differences were noted, including a higher rate of virus shedding in oral secretions and a higher incidence of focal hepatic necrosis in NiV_B_-infected ferrets, whereas there was a higher viral load in the blood at time of euthanasia and a higher rate of hemorrhagic diathesis in the NiV_M_-infected ferrets. Interestingly, in contrast to the hamster model, the higher rate of virus shedding in oral secretions in NiV_B_-infected ferrets suggested a potential mechanism for the higher rate of human-to-human transmission in NiV_B_-infected humans.

In the current study we compared the NiV_M_ and NiV_B_ strains with similar passage history in AGMs. The initial report on the NiV_M_ model in AGMs was reported as nearly uniformly lethal with various routes of infection and doses being examined and 7 of 8 AGMs succumbing. Subsequent studies have revealed the model to be less lethal than initially thought, depending on dose and route of infection^13^,^35^,^36^,^37^,^38^,^39^,^40^. When these studies are combined, all animals challenged with various combinations of i.t, i.n., intraperitoneal, and/or oral routes with various doses have only had 12 out of 19 (~63% lethality) total AGMs succumb, although all AGMs did show clinical disease. The lethality does not appear to be dependent on the various challenge doses used in these studies.

In the present study we challenged AGMs under identical conditions; virus passage history/cells, dose of inoculum, and route of inoculation. We found that AGMs challenged with NiV_B_ resulted in 100% mortality compared to 50% for NiV_M_, at least within the 15 day window of acute, respiratory-mediated disease; within these constraints we found the survival curves to be significantly different (Fig. 1c, *). We cannot rule out the possibility that the surviving AGMs infected with NiV_M_ would not have succumbed later due to late-onset neurological disease, although some survivors in the previously mentioned studies survived to day 32 without developing neurological disease^40^. In addition to the 6 AGMs infected with NiV_B_ presented in the current study, we have 4 additional AGMs which have been infected with NiV_B_ under identical conditions presented in the current study although at three different times (unpublished data). These animals all succumbed between 5–9 days p.i. Thus, including historical data, we see 10 of 10 AGMs infected with NiV_B_ succumbing with only 12 of 19 AGMs infected with NiV_M_ succumbing. Additionally, from these unpublished data, historical data, and the present study we see AGMs infected with NiV_B_ succumbing between days 5–9, while AGMs infected with NiV_M_ succumb between days 9–12. Specifically, in this current study, NiV_B_ had a shortened time to death in AGMs by an average of 2.8 days; NiV_B_ produced higher titers in target human pulmonary endothelial cell lines and primary cells, and higher virus load in the lungs and oral swabs of infected AGMs. A higher NiV_B_ virus load was also observed in the blood, lymphoid tissue, and liver in AGMs when compared to NiV_M_-infected AGMs. These data together suggest that there is an intrinsic difference between the two strains of NiV, particularly as the AGM model recapitulated the more severe respiratory disease and significant histopathology differences, shorter time to death, and higher mortality observed in NiV_B_ human outbreaks when compared to outbreaks of NiV_M_. These differences require further characterization to elucidate which proteins and/or genomic elements are responsible for NiV_B_ having a more acute and increased respiratory disease.

Recently we reported the 100% successful therapeutic human mAb m102.4 treatment of AGMs infected with NiV_M_ showing clinical signs of disease along with detectable viremia^37^. Additionally, m102.4 was employed in 2013 in the United States to treat a possible laboratory exposure to NiV_B_ under a compassionate use protocol^41^. This individual has never shown any evidence of henipavirus infection. Although this individual did not experience any NiV_B_ disease manifestations considering the shortened disease course and higher viremia in the NiV_B_ AGM model, we were interested in determining whether or not the therapeutic treatment window with m102.4 was similar to the NiV_M_ model. Importantly, we found that the treatment window for NiV_B_ is shorter as the D5/D7 treatment group succumbed to NiV_B_-mediated disease whereas this treatment regimen was completely protective against NiV_M_-mediated disease in AGMs with a significant difference in survival curve observed between the D5/D7 treatment groups^37^. All AGMs in the D3/D5 group seroconverted in the NiV_B_ study whereas only 1 AGM out of 4 in the NiV_M_ D3/D5 study seroconverted. The average D5/D7 reciprocal neutralizing titers on day 7 NiV_B_ post-challenge were much lower (426.6) compared to the NiV_M_ study (5120) as well. All of these observed differences are most likely due to the more rapid and higher viral loads experienced by AGMs after exposure to NiV_B_ versus NiV_M_ but should be considered when assessing treatment options between someone exposed to NiV_M_ or NiV_B_.

The exact mechanism of treatment failure in the D5/D7 group is not clear. The half-life of m102.4 in AGMs ranges from 10–12 days^42^ and therefore is not the cause of treatment failure in the D5/D7 group. Rather, the D5/D7 group had a noticeably decreased level of neutralizing antibody of the exogenously infused m102.4 compared to the other cohorts (320–640 compared to 1280–2560) at the time point 2 days after the first infusion (Supplementary Table 2). This may be due to higher loads of circulating virus on the day of initial treatment being bound by the m102.4, although since there was one animal (O8149) in the Day 3/5 group with a similarly high level of viremia on the day of its first treatment; that animal survived and did not have a low neutralizing titer. Perhaps the significant differences observed in the spleen histopathology between NiV_M_ and NiV_B_ (Fig. 5b) could account for this as these cells and germinal centers would also be needed to fully recover during a therapeutic treatment study. Alternatively, it may be due to loss of m102.4 through the leaky capillaries in the D5/D7 group as observed by the hypoalbuminemia observed in this group (Table 2). It is unknown if a higher dose of m102.4 treatments may have overcome these potential reasons for treatment failure in the D5/D7 group. There are however, at least two other considerations for the failure of the treatment for NiV_B_. We found no changes in the amino acids involved in the m102.4 binding pocket of the NiV glycoprotein as these are completely conserved between all isolates of NiV_B_ and NiV_M_ examined previously^43^ as well as in the isolates used in this study (Supplementary Table 1). Additionally, escape mutants were not the cause as we were unable to isolate any escape mutants from the blood or tissues recovered from the D5/D7 group at the terminal time points. Regardless of the reason for the D5/D7 treatment failure, we have shown that the efficacious treatment window is shorter for NiV_B_ in AGMs which has implications for how aggressive treatment initiation should be undertaken after exposure to NiV_B_ as it is often difficult for decisions on what treatments should be given for drugs that do not have licensure.

## Methods

### Virus isolates

The isolate of NiV_M_ used in the pathogenic comparison study was 199902916 and was obtained from a fatal human case in 1999 and passaged on Vero E6 cells twice making this a passage 2 virus. The isolate of NiV_B_ used in the pathogenic comparison and m102.4 study was 200401066 and was obtained from a fatal human case during the outbreak in Rajbari, Bangladesh in 2004 and passaged on Vero E6 cells twice making this a passage 2 virus. Both strains of NiV used in this study were kindly provided by Dr. Thomas G. Ksiazek. Each NiV challenge virus stock was assessed for the presence of endotoxin using The Endosafe^®^-Portable Test System (PTS) (Charles River). Virus preparations were diluted 1:10 in Limulus Amebocyte Lysate (LAL) Reagent Water (LRW) per manufacturer’s directions and endotoxin levels were tested in LAL Endosafe^®^-PTS cartridges as directed by the manufacturer. Each preparation was found to be below detectable limits while positive controls showed that the tests were valid.

Approximately 1 ml of NiV stock was removed from the seed vial and placed in 5 ml of Trizol LS and vortexed 3 times and allowed to sit for 10 minutes. The 6 ml were then placed into 2 separate 3 ml Nunc cryo-vials for removal from the BSL-4. RNA was isolated from the Trizol LS/sample mixture using Zymo Research Direct-zol RNA mini-prep per manufacturer’s instructions. Approximately 150 ng of purified RNA were used to make cDNA using the NuGen Ovation RNA-seq 2.0 kit ultimately for the preparation of the double stranded DNA library using Encore Ion Torrent library prep kit. Sequencing was performed by the UTMB Molecular Core on the Ion Torrent using 318-v2 deep sequencing chips. Sequence analysis was performed using DNA Star Seqman NGen software based on unpaired analysis of 125 bp overlaps.

### Endothelial cell culture methods and NiV infection

The human brain microvascular endothelial cell line, hCMEC/D3, was a generous gift from Dr. Babette Weksler (Cornell, Ithica, NY, USA) and was maintained in EBM-2 media (Lonza, Walkersville, MD, USA), containing 5% fetal bovine serum, 1.4 μM hydrocortisone, 5 μg/ml absorbic acid, 1 ng/ml basic fibroblast growth factor (Sigma), 1X chemically defined lipid concentrate, 10 mM 4-(2-hydroxyethyl)-1-piperazineethanesulfonic acid (HEPES), and Penicillin-Streptomycin (Life Technologies)^44^. Cells were grown in cell culture vessels precoated with gelatin (0.2%). Media was changed every 2–3 days.

The human lung microvascular endothelial cell line, hpmecst1.6r, was a generous gift from Drs Unger and Kirkpatrick (Johannes-Gutenberg University, Mainz, Germany). These cells were maintained in medium containing M199, 20% FCS, Glutamax (2 mm), Penicillin-Streptomycin (100U/100 μg/ml), heparin (50 μg/ml), and ECGS (50 μg/ml) and G418 (50 μg/ml, (Life Technologies). Cells were grown in cell culture vessels pre-coated with gelatin (0.2%). Media was changed every 2–3 days^45^.

Human pulmonary microvascular endothelial cells (HPMEC) were maintained in EBM-2 MV media supplemented with 5% fetal bovine serum and proprietary concentrations of human epidermal growth factor, fibroblast growth factor, vascular endothelial growth factor, insulin-like growth factor, hydrocortisone, and gentamicin all provided by Lonza. Human brain cerebrum microvascular endothelial cells (HBCMEC) were obtained from Sciencell and maintained in EBM-2 containing 5% fetal bovine serum, 1.4 μM hydrocortisone, 5 ug/ml absorbic acid, 1 ng/ml basic fibroblast growth factor (Sigma, St. Louis, MO, USA), 1X chemically defined lipid concentrate, 10 mM HEPES, and Penicillin-Streptomycin (Life Technologies). Media was changed every 2–3 days and cells were used up to passage 7.

To assess the growth kinetics of both NiV strains in target cells, ECs were infected with NiV_M_ or NiV_B_ at a MOI of 5 (MOI of 1 for primary cells) with rocking for 1 hour at 37 °C. Inoculum was removed, cells were washed, and appropriate media (described above) was added back to cells. Samples were taken from initial addition of media at 1 hour post-infection (hpi) as a baseline and from the same wells at 24 and 48 hpi.

### Ethics statement

Healthy, adult AGMs were handled in the animal BSL-4 containment space at the Galveston National Laboratory (GNL), Galveston, Texas. Research was approved by the University of Texas Medical Branch (UTMB) Institutional Animal Care and Use Committee (IACUC). This facility is fully accredited by the Association for Assessment and Accreditation of Laboratory Animal Care International. All methods were performed in accordance with relevant guidelines and regulations. Animals were housed in adjoining individual primate cages allowing social interactions, under controlled conditions of humidity, temperature, and light (12-hour light/12-hour dark cycles). Food and water were available ad libitum. Animals were monitored (pre- and post-infection) and fed commercial monkey chow, treats and fruit twice daily by trained personnel. Environmental enrichment consisted of commercial toys. All procedures were conducted by trained personnel under the oversight of an attending veterinarian and all invasive clinical procedures were performed while animals were anesthetized using ketamine. Animals were euthanized using a pentobarbital-based euthanasia solution.

### Pathogenesis Challenge

Eight, adult, AGMs weighing 4 to 6 kg were used in this study and were randomized into two groups of 4 animals each (3 males and 1 female per group). Animals were inoculated with ~5 × 10^5^ PFU of either NiV_M_ or NiV_B_ with the dose being equally divided between the intratracheal (i.t.) and the intranasal (i.n.) routes for each animal. After challenge, animals were monitored for clinical signs of illness including temperature, respiration quality, blood count, and clinical pathology on days 0, 1, 3, 5, 7, 10, and 15 post-challenge.

### Challenge and m102.4 Treatment

Eleven adult AGMs weighing 4–8 kg were inoculated by i.t. and i.n. routes with ~5 × 10^5^ PFU of NiV_B_ as above. Three animals were infused with m102.4 beginning 1 day after challenge and again 3 days after challenge (D1/D3); three animals were infused with m102.4 beginning 3 days after challenge and again 5 days after challenge (D3/D5); three animals were infused with m102.4 beginning 5 days after challenge and again 7 days after challenge (D5/D7). The two control animals were infused with saline. Each dose of m102.4 (~15 mg/kg) was administered intravenous (i.v.) Animals were anesthetized for antibody infusion and clinical examination including temperature, respiration rate/quality, blood collection, and swabs of nasal, oral, and rectal mucosa on days 0, 3, 5, 7, 10, 15, 21, and 28 post-challenge.

### Hematology and Serum Biochemistry

Hematological analysis including total white blood cell counts, white blood cell differentials, red blood cell counts, platelet counts, hematocrit values, total hemoglobin concentrations, mean cell volumes, mean corpuscular volumes, and mean corpuscular hemoglobin concentrations were analyzed from blood collected in tubes containing EDTA using a laser based hematologic analyzer (Beckman Coulter). Serum samples were tested for concentrations of albumin, amylase, alanine aminotransferase (ALT) aspartate aminotransferase (AST), alkaline phosphatase (ALP), gamma-glutamyltransferase (GGT), glucose, cholesterol, total protein, total bilirubin (TBIL), blood urea nitrogen (BUN), creatinine (CRE), and C-reactive protein (CRP) by using a Piccolo point-of-care analyzer and Biochemistry Panel Plus analyzer discs (Abaxis).

### RNA Isolation

Immediately following sampling, 100 μl of blood was added to 600 μl of AVL viral lysis buffer (Qiagen) for RNA extraction. For tissues, approximately 100 mg was stored in 1 ml RNAlater (Qiagen) for 7 days to stabilize RNA. RNAlater was completely removed, and tissues were homogenized in 600 μl RLT buffer (Qiagen) in a 2-mL cryovial using a tissue lyser (Qiagen) and ceramic beads. The tissues sampled included conjunctiva, tonsil, oro/nasopharynx, nasal mucosa, trachea, right bronchus, left bronchus, right lung upper lobe, right lung middle lobe, right lung lower lobe, right lung upper lobe, right lung middle lobe, right lung lower lobe, bronchial lymph node (LN), heart, liver, spleen, kidney, adrenal gland, pancreas, jejunum, colon transversum, brachial plexus, brain (frontal and cerebellum), brain stem, cervical spinal cord, pituitary gland, mandibular LN, salivary gland LN, inguinal LN, axillary LN, mesenteric LN, urinary bladder, testes or ovaries, and femoral bone marrow. All blood samples were inactivated in AVL viral lysis buffer, and tissue samples were homogenized and inactivated in RLT buffer prior to removal from the BSL-4 laboratory. Subsequently, RNA was isolated from blood and swabs using the QIAamp viral RNA kit (Qiagen), and from tissues using the RNeasy minikit (Qiagen) according to the manufacturer’s instructions supplied with each kit.

### Detection of NiV load

RNA was isolated from blood or tissues and analyzed using strain specific primers/probe targeting the N gene and intergenic region between N and P of NiV_M_ or NiV_B_ for quantitative real-time PCR (qRT-PCR) with the probes used here being 6-carboxyfluorescein (6FAM)-5′ CGT CAC ACA TCA GCT CTG ACG A 3′-6 carboxytetramethylrhodamine (TAMRA) for NiV_M_ and 6FAM-5′CGT CAC ACA TCA GCT CTG ACA A 3′-6TAMRA for NiV_B_ (Life Technologies, Carlsbad, CA). This strategy using the intergenic region allows for genome and anti-genome detection only without detecting contaminating viral mRNA. NiV RNA was detected using the CFX96 detection system (Bio-Rad) in One-step probe qRT-PCR kits (Qiagen) with the following cycle conditions: 50 °C for 10 minutes, 95 °C for 10 seconds, and 40 cycles of 95 °C for 10 seconds and 59 °C for 30 seconds. Threshold cycle (*CT*) values representing NiV genomes were analyzed with CFX Manager Software, and data are shown as genome equivalents (GEq). To create the GEq standard, RNA from NiV challenge stocks was extracted and the number of NiV genomes was calculated using Avogadro’s number and the molecular weight of the NiV genome.

Virus titration was performed by plaque assay with Vero cells from all blood and tissue samples. Briefly, increasing 10-fold dilutions of the samples were adsorbed to Vero cell monolayers in duplicate wells (200 μl); the limit of detection was 25 PFU/ml.

### Histopathology and immunohistochemistry analyses and scoring

Necropsy was performed on all subjects. Tissue samples of all major organs were collected for histopathologic and immunohistochemical examination and were immersion-fixed in 10% neutral buffered formalin for at least 21 days in BSL-4. Subsequently, formalin was changed; specimens were removed from BSL-4, processed in BSL-2 by conventional methods and embedded in paraffin and sectioned at 5 μm thickness. For immunohistochemistry, specific anti-NiV immunoreactivity was detected using an anti-NiV N protein rabbit primary antibody at a 1:5000 dilution for 30 minutes. The tissue sections were processed for immunohistochemistry using the Dako Autostainer (Dako). Secondary antibody used was biotinylated goat anti-rabbit IgG (Vector Laboratories, Burlingame, CA) at 1:200 for 30 minutes followed by Dako LSAB2 streptavidin-HRP (Dako) for 15 minutes. Slides were developed with Dako DAB chromagen (Dako) for 5 minutes and counterstained with hematoxylin for one minute. Non-immune rabbit IgG was used as a negative staining control.

To assess the differences in histopathology Principles for Valid Histopathologic Scoring in Research^46^ were used where a scoring system was 1) defined 2) repeatable and 3) produced meaningful results. The tissues for the study were group masked and scored in an ordinal scoring fashion, meaning that samples were assigned to a category in an ordered progression in severity; such as, 0 to 4 for immunohistochemistry and 0 to 5 on H&E based on an estimated percentage of the organ showing the lesion. For the ordinal scoring in this study the median was assessed as this is more appropriate when recording the central tendency. The slide sets were reviewed by one pathologist multiple times to reduce any interpretation consistency. Median values were calculated on selected organs to record the central tendency. Scoring index used was based on percent affected tissue: 0- no lesion (none); 1- minimal change (10% and less); 2- mild change (11–25%); 3- moderate change (26–50%); 4- marked change (51–75%); 5- severe change (76–100%).

### NiV_B_ serum neutralization assays

Neutralization titers against NiV_B_ were determined by a conventional serum neutralization assay. Briefly, m102.4 or sera were serially diluted fivefold or twofold respectively, and incubated with ~100 PFU of NiV_B_ for 1 h at 37 °C. Virus and antibodies mixtures were then added to individual wells of 6-well plates of Vero cells. Plates were stained with neutral red 2 days after infection and plaques were counted 24 h after staining. The 50% neutralization titer was determined as the serum dilution at which at there was a 50% reduction in plaque counts versus control wells.

### Measurement of circulating F glycoprotein specific antibodies

Antibodies to the fusion (F) glycoprotein were measured in NiV_B_-infected AGMs by including a recombinant soluble F (sF) glycoprotein-coupled microsphere in the assay by coupling of sF to microsphere #43 (Luminex Corporation)^42^. Plasma from NiV_B_-infected AGMs was inactivated by gamma irradiation (~5 mrad) prior to testing. Sera and plasma were assayed at 1:5,000 and 1:10,000 dilutions. Assays were performed on a Luminex^®^ 200 IS™ machine equipped with Bio-Plex Manager Software (v 5.0) (Bio-Rad Laboratories). Mean fluorescent intensity (M.F.I.) and the standard deviation of fluorescence intensity across 100 beads were determined for each sample.

### Statistics

Conducting animal studies in BSL-4 severely restricts the number of animal subjects, the volume of biological samples that can be obtained and the ability to repeat assays independently and thus limit statistical analysis. Consequently, data are presented as the mean calculated from replicate samples, not replicate assays, and error bars represent the standard deviation across replicates.

Prism 5 software was used to calculate statistical significance throughout this study to perform ANOVA with Dunnett’s Multiple Comparison Test for viral growth kinetics, m102.4 neutralization, and viral load data; Log-rank (Mantel-Cox) Test for Kaplan-Meier survival curves; Mann-Whitney test for ordinal scoring of tissue pathology.

## Additional Information

**How to cite this article**: Mire, C. E. *et al*. Pathogenic Differences between Nipah Virus Bangladesh and Malaysia Strains in Primates: Implications for Antibody Therapy. *Sci. Rep*. **6**, 30916; doi: 10.1038/srep30916 (2016).

## Supplementary Material

Supplementary Information

## Figures and Tables

**Table 1:** Clinical findings and outcome for AGMs challenged with NiV.

Subject No.	Sex	NiV strain	Viral load^a^	Clinical illness^c^	Clinical and gross pathology^d^
O7912^	Male	Malaysia	Ø^b^	Fever (d7); Depression (d7–11); Loss of appetite (d7–11); Dyspnea (d7–12); Labored breathing (d7–12); Nasal exudate (d9).	Thrombocytopenia (d10); Lymphopenia (d7); Hypoalbuminemia (d10,15); >2-fold increase in ALT (d5); Increase in CRP (d7,15); Moderate congestion at gastroduodenal junction.
O7935^	Female	Malaysia	Ø	Fever (d7); Depression (d7–12); Loss of appetite (d7–13); Dyspnea (d7–12); Labored breathing (d7–12); Lethargy (d9–12); Tremors (d9–13); Nasal exudate (d5).	Thrombocytopenia (d10); Lymphopenia (d6,8); Hypoalbuminemia (d10,15); Increase in CRP (d7,10).
O8000	Male	Malaysia	Ø	Fever (d7); Depression (d7–10); Loss of appetite (d7–10); Dyspnea (d7–10); Labored breathing (d7–10); Lethargy (d10); Tremors (d10); Nasal exudate (d10); Epistaxis (d10). Animal euthanized d10.	Thrombocytopenia(d10); Lymphopenia (d7); Hypoalbuminemia (d10); Increase in CRP (d7,10); Congestion of nares; Excess fluid in the pleural cavity; Lungs inflated and enlarged with multifocal areas of congestion and hemorrhage; Enlarged spleen darkened and mottled; Liver slightly darkened and reticulated; kidney pale and reticulated; Stomach with mild multifocal areas of congestion and small petechia on mucosal surface; Mild congestion at gastroduodenal junction; Prominent hemorrhages on mucosal surface of urinary bladder.
O8023	Male	Malaysia	d7	Fever (d7); Depression (d7–10); Loss of appetite (d7–10); Dyspnea (d7–10); Labored breathing (d7–10); Lethargy (d9–10). Animal euthanized d10.	Thrombocytopenia (d5,7,10); Lymphopenia (d7); Hypoalbuminemia (d10); >2-fold increase in AST (d7); >3-fold increase in AST (d10); Increase in CRP (d7,10); Excess fluid in the pleural cavity; Lungs inflated and enlarged with multifocal areas of congestion and hemorrhage; Enlarged spleen darkened and mottled; Liver slightly darkened; Kidney pale and reticulated; Stomach with mild multifocal areas of congestion and small petechia on mucosal surface; Mild congestion at gastroduodenal junction; Hemorrhages on mucosal surface of urinary bladder; Congestion of small blood vessels in the brain.
O8021*	Male	Bangladesh	d5	Loss of appetite (d4–7); Dyspnea (d7); Labored breathing (d6–7). Animal succumbed d7.	Lymphopenia (d5); Excess fluid in the pleural cavity; Lungs inflated and enlarged with multifocal areas of congestion and hemorrhage; Enlarged spleen darkened and mottled; Liver darkened and reticulated; Kidney pale and reticulated; Stomach with multifocal areas of congestion and small petechia on mucosal surface; Hemorrhages on mucosal surface of urinary bladder; Congestion of small blood vessels in the brain.
O7936*	Female	Bangladesh	d5	Loss of appetite (d6–7). Animal succumbed d7.	Thrombocytopenia (d5); Lungs inflated and enlarged with multifocal areas of congestion and hemorrhage; spleen darkened and mottled; liver darkened and reticulated; kidney pale and reticulated; hemorrhages on mucosal surface of urinary bladder.
O8022	Male	Bangladesh	d5,7	Fever (d7); Depression (d7); Loss of appetite (d7); Dyspnea (d7); Labored breathing (d7); Lethargy (d7); Nasal exudate (d7); Epistaxis (d7). Animal succumbed d7.	Thrombocytopenia (d5,7); Lymphopenia (d7); Hypoalbuminemia (d7); >2-fold increase in ALT (d5); >2-fold increase in AST (d5,7); >2-fold increase in BUN (d7); Increase in CRP (d7); Frothy nasal exudate; Excess fluid in the pleural cavity; Lungs inflated and enlarged with multifocal areas of congestion and hemorrhage; Spleen darkened and mottled; Liver darkened and reticulated; Kidney pale and reticulated; Stomach with multifocal areas of congestion and small petechia on mucosal surface; Hemorrhages on mucosal surface of urinary bladder.
O8046	Male	Bangladesh	d5,7	Fever (d7); Depression (d7); Loss of appetite (d7); Dyspnea (d7); Labored breathing (d7); Lethargy (d7); Nasal exudate (d6–7); Tremors (d7). Animal succumbed d7.	Thrombocytopenia (d7); Lymphopenia (d7); Hypoalbuminemia (d7); >2-fold increase in AST (d5,7); Increase in CRP (d7); Frothy sanguineous nasal exudates; Excess fluid in the pleural cavity; Lungs inflated and enlarged with multifocal areas of congestion and hemorrhage; Spleen darkened and mottled; Liver darkened and reticulated; Kidney pale and reticulated; Stomach with multifocal areas of congestion and small petechia on mucosal surface; Hemorrhages on mucosal surface of urinary bladder.

^^^Survived to day 15.

^*^O7936 and O8021 succumbed before sampling at day 7 could be achieved.

^a^Infectious virus isolated from plasma on day(s) indicated.

^b^Below limit of detection.

^c^Days after NiV challenge are in parentheses. Fever is defined as a temperature more than 2.5 ^°^F over baseline or at least 1.5 ^°^F over baseline and ≥103.5 ^°^F.

^d^Lymphopenia and thrombocytopenia are defined by a ≥30% drop in numbers of lymphocytes and platelets, respectively. ALT- alanine aminotransferase; CRP- C reactive protein; AST- aspartate aminotransferase; BUN- blood urea nitrogen.

**Table 2:** Clinical Description and Outcome of NiV_B_-Challenged and m102.4 Treated AGMs.

Subject No.	Sex	Group	Clinical illness	Clinical and gross pathology
O7923	Male	Control	Fever (d7); Depression (d7–8); Lethargy (d7–8); Loss of appetite (d6–8); Dyspnea (d8); Labored breathing (d5–8). Animal euthanized on d8.	Thrombocytopenia (d5,7,8); Lymphopenia (d5,7) Hypoalbuminemia (d8); >7-fold increase in ALT (d8); >2-fold increase in AST (d5,7); >10-fold increase in AST (d8); 2-fold increase in BUN (d8); >4-fold increase in CRE (d8); Increase in CRP (d7–8); Excess fluid in the pleural cavity; Lungs inflated and enlarged with coalescing multifocal areas of congestion and hemorrhage; Excess pericardial fluid; Enlarged adrenal glands; Liver darkened and reticulated; Congestion on mucosal surface of urinary bladder; Congestion of small blood vessels in the brain.
C4373	Male	Control	Depression (d7); Lethargy (d7); Loss of appetite (d5–7); Severe dyspnea (d7); Labored breathing (d5–7); Nasal exudates (d7); Epistaxis (d7). Animal euthanized on d7.	Thrombocytopenia (d7); Lymphopenia (d3,5,7); Hypoabluminemia (d7); >2-fold increase in AST (d5); >3-fold increase in AST (d7); Increase in CRP (d 5, 7); Excess fluid in the pleural cavity; Lungs inflated and enlarged with multifocal areas of congestion and hemorrhage; Extensive fibrin mats along pleural surfaces; Liver darkened and reticulated; Multifocal areas of hemorrhage on mucosal surface of urinary bladder; Congestion of meninges in the brain.
O7698	Female	D1/D3	Mild abdominal breathing (d6–7); Mild dyspnea (d7).	None
O8215	Male	D1/D3	None	>2-fold increase in ALT (d5); >2-fold increase in AST (d7).
O8214	Male	D1/D3	Mild abdominal breathing (d6).	None
O7522	Male	D3/D5	None	Lymphopenia (d5,7); >2-fold increase ALT (d7); >2-fold increase AST (d7).
O8149	Female	D3/D5	None	Lymphopenia (d3,d7); Increase in CRP (d7).
O8211	Male	D3/D5	None	Lymphopenia (d7); >3-fold increase in AST (d5); >2-fold increase in AST (d7).
B4825	Male	D5/D7	Fever (d7); Depression (8); Loss of appetite (d7–8); Dyspnea (d7–8); Labored breathing (d5–8); Tremors (d8). Animal euthanized on d8.	Thrombocytopenia (d7,8); Lymphopenia (d7); Hypoalbuminemia (d7,8); >2-fold increase in AST (d5); >3-fold increase in AST (d7); >10-fold increase in AST (d8); Increase in CRP (d5,7,8); Excess fluid in the pleural cavity; Lungs inflated and enlarged with multifocal areas of congestion and hemorrhage; Fibrin mats along pleural surfaces; Liver darkened and reticulated; Congestion of meninges in the brain.
B6484	Male	D5/D7	Fever (d7); Depression (d8); Loss of appetite (d6–8); Dyspnea (d5–8), Labored breathing (d5–8); Nasal exudates (d8). Animal euthanized on d8.	Thrombocytopenia (d8); Lymphopenia (d3,5,7); Hypoalbuminemia (d8); >2-fold increase in ALT (d5,7,8); >2-fold increase in AST (d5,7); >3-fold increase in AST (d8); >2-fold increase in BUN (d8); Increase in CRP (d7, 8); excess fluid in the pleural cavity; Lungs inflated and enlarged with multifocal areas of congestion and hemorrhage; Minimal fibrin mats along pleural surfaces; Congestion and fluid within the meninges of the brain.
C3882	Male	D5/D7	Fever (d7); Depression (d8); Loss of appetite (d6–8); Dyspnea (d5 and 8); Labored breathing (d5–8); Tremors (d8). Animal succumbed d8.	Thrombocytopenia (d3,7,8); Hypoalbuminemia (d8); >4-fold increase in AST (d8); >2-fold increase in BUN (d8); Increase in CRP (d7,8); Excess fluid in the pleural cavity; Lungs inflated and enlarged with multifocal areas of congestion and hemorrhage; Extensive fibrin mats along pleural surfaces; Liver darkened and reticulated; Multifocal areas of hemorrhage on mucosal surface of urinary bladder; Congestion of meninges in the brain.
