# Supplementary material for: Pathogenic Differences between Nipah Virus Bangladesh and Malaysia Strains in Primates: Implications for Antibody Therapy
Source: Sci Rep. 2016 Aug 3;6:30916. doi: 10.1038/srep30916 (PMC4971471; doi:10.1038/srep30916)
Supplement: Supplementary Information [file srep30916-s1.doc]

**TITLE**

**Pathogenic Differences between Nipah Virus Bangladesh and Malaysia Strains in Primates: Implications for Antibody Therapy**

**AUTHORS AND AFFILIATIONS**

Chad E. Mire^1,2^, Benjamin A. Satterfield^1,2^, Joan B. Geisbert^1,2^, Krystle N. Agans^1,2^, Viktoriya Borisevich^1,2^, Lianying Yan^3^, Yee-Peng Chan^3^, Robert W. Cross^1,2^, Karla A. Fenton^1,2^, Christopher C. Broder^3^, and Thomas W. Geisbert^1,2*^

^1^Galveston National Laboratory and ^2^Department of Microbiology and Immunology, University of Texas Medical Branch, Galveston, TX, USA, ^3^Department of Microbiology and Immunology, Uniformed Services University of the Health Sciences, Bethesda, Maryland, USA

^*^Corresponding author: [twgeisbe@utmb.edu](mailto:twgeisbe@utmb.edu)

**
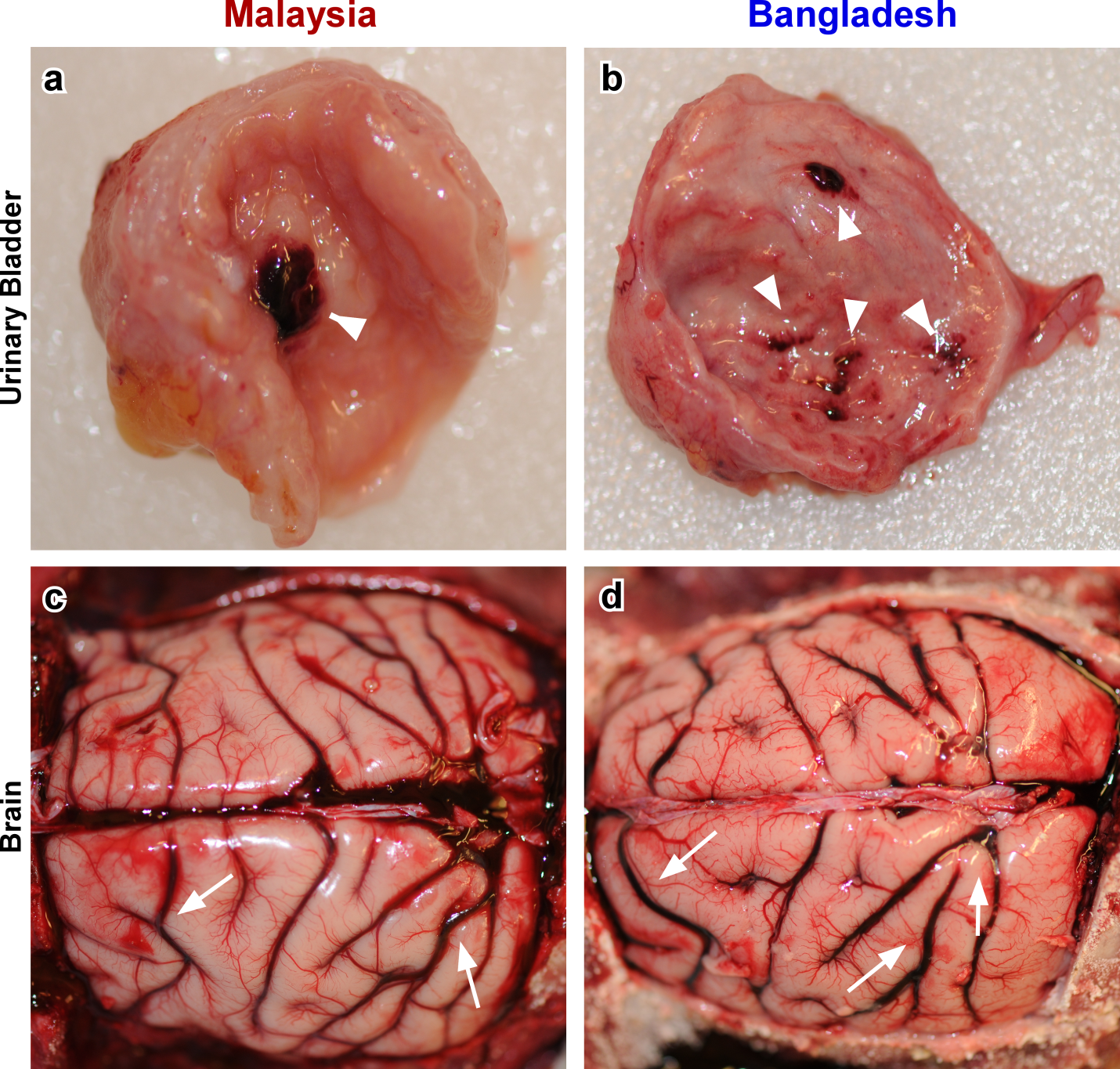
**

**Supplementary Figure 1. Gross pathology of urinary bladders and brains of NiV_M_ and NiV_B_ infected AGMs.** Representative hemorrhage (white arrowheads) on the mucosal surface of the urinary bladder of AGMs infected with NiV_M_ (a) and NiV_B_ (b). Representative vascular congestion (white arrows) on the surface of the AGM brains after infection with NiV_M_ (c) and NiV_B_ (d).


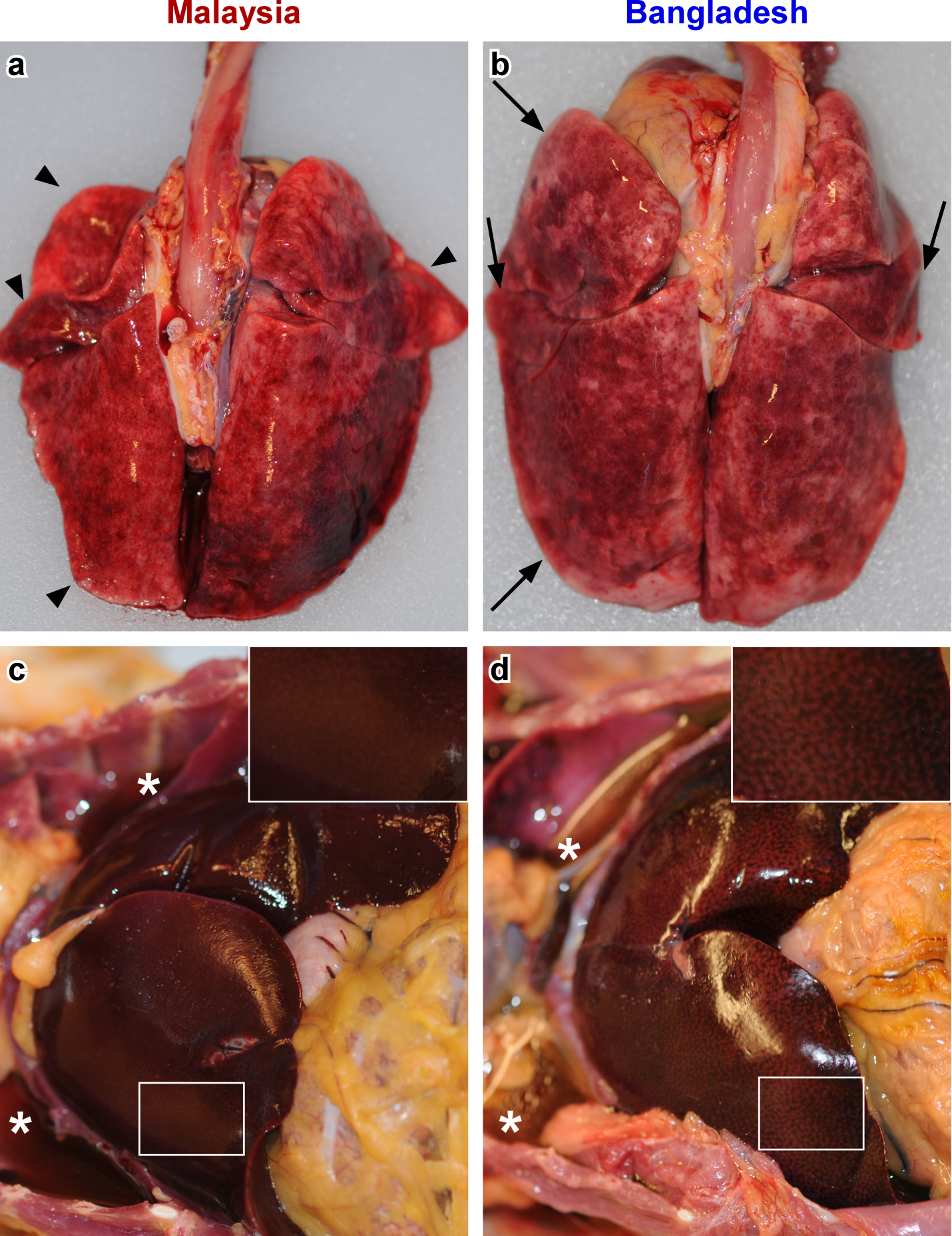


**Supplementary Figure 2. Gross pathology of lungs and livers of NiV_M_ and NiV_B_ infected AGMs.** Representative gross pathology of lungs with NiV-induced coalescing hemorrhagic and necrotic foci induced by NiV_M_ (a) and NiV_B_ (b). The NiV_M_ lung lobes (a) have defined edges (arrowheads) representative of collapsed lobes while the NiV_B_ lung lobes (b) have more rounded, less defined edges (arrows) representative of edema inflated lungs. Comparison of the representative liver gross pathology between NiV_M_ (c) and NiV_B_ (d) showing a marked nutmeg pattern in the inset in (d) as compared to (c). White asterisks mark serosanguinous fluid in the pleural cavity.

**
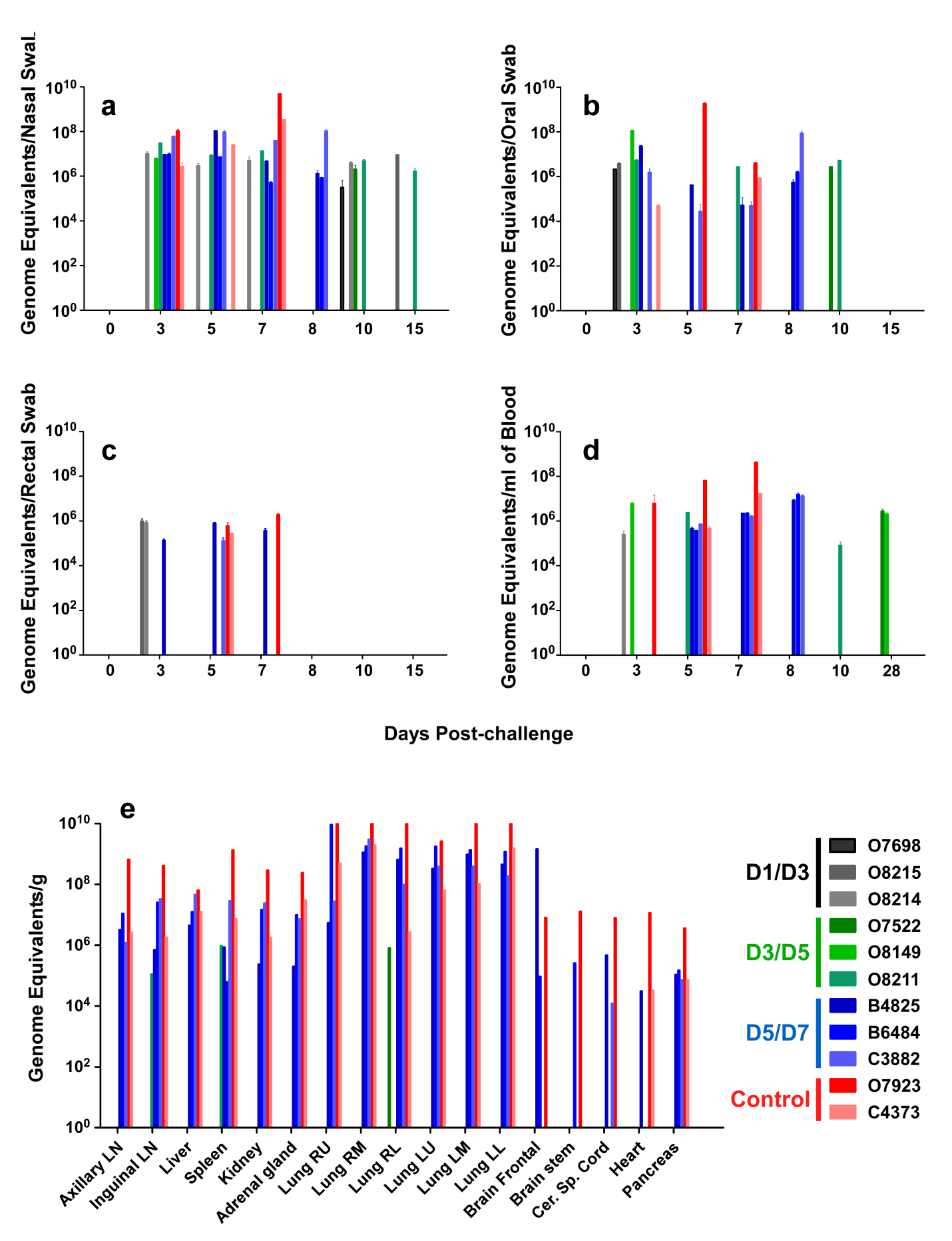
**

**Supplementary Figure 3. Viral loads in m102.4 treated AGMS.** NiV_B_ viral RNA genomic equivalents detected by qRT-PCR from AGM nasal swabs (a), oral swabs (b), rectal swabs (c), circulating in blood (d) and by genome equivalents/g of tissue (e). Group D1/D3 (gray); Group D3/D5 (green); Group D5/D7 (blue); and control Group (red). LN (lymph node), RU (right upper), RM (right middle), RL (right lower), LU (left upper), LM (left middle), LL (left lower), Cer. Sp. (cervical spinal). Error bars represent standard error of the mean.

| **Supplementary Table 1. SNP changes from deep sequencing data.** | | | | | | | | | | | |
| --- | --- | --- | --- | --- | --- | --- | --- | --- | --- | --- | --- |
|  | | | | | | | | | | | |
| **NiV-Malaysia P2^a^** | | | | | | | | | | | |
| **Ref Pos** | **Type** | **Ref Base^b^** | **Called Base** | **SNP %** | **Depth** | **A Cnt** | **C Cnt** | **G Cnt** | **U Cnt** | **Gene** | **Change** |
| 520 | SNP | A | G | 100.00% | 1000 | - | 0 | 1000 | 0 | N | Silent |
| 1596 | SNP | A | G | 99.80% | 1000 | - | 0 | 998 | 0 | N | N495S |
| 5602 | SNP | T | C | 99.60% | 1000 | 0 | 996 | 0 | - | M | Silent |
| 6461 | SNP | A | C|U | 99.00% | 1000 | - | 990 | 0 | 3 |  | non-coding |
| 7211 | SNP | A | G|U | 99.30% | 1000 | - | 1 | 993 | 0 | F | Silent |
| 9485 | SNP | A | G|U | 99.50% | 984 | - | 0 | 979 | 1 | G | Silent |
| 10932 | SNP | A | G|U | 99.80% | 521 | - | 0 | 520 | 1 |  | non-coding |
| 14495 | SNP | T | C|A|G | 97.60% | 1000 | 3 | 976 | 1 | - | L | Silent |
| 16037 | SNP | T | C|A | 98.60% | 1000 | 4 | 986 | 0 | - | L | Silent |
| 17897 | SNP | A | G | 96.10% | 1000 | - | 0 | 961 | 0 | L | Silent |
| **NiV-Malaysia P3^c^** | | | | | | | | | | | |
| 9733 | SNP | C | C|A|G | 15.70% | 1000 | 157 | - | 1 | 1 | G | N277K |
| 16607 | SNP | A | G|A|U | 12.80% | 1000 | - | 10 | 128 | 32 | L | Silent |
| **NiV-Bangladesh P2^d^** | | | | | | | | | | | |
| **Ref Pos** | **Type** | **Ref Base^e^** | **Called Base** | **SNP %** | **Depth** | **A Cnt** | **C Cnt** | **G Cnt** | **U Cnt** | **Gene** | **Change** |
| 2099 | SNP | T | A|G | 11.10% | 1000 | 111 | 0 | 1 | - |  | non-coding |
| 5118 | SNP | A | G | 10.80% | 1000 | - | 0 | 108 | 0 | M | D4G |
| 7279 | SNP | C | U | 22.10% | 1000 | 0 | - | 0 | 221 | F | S207F |
| 7414 | SNP | G | A | 99.10% | 1000 | 991 | 0 | - | 0 | F | G252D |
| The binding sites for m102.4 to the NiV G protein include S239, C240, S241, R242, L305, F458, Q490, W504, E505, V507, Q530, E533, D555, N557, Q559, Y581, I588. As described previously^38^. All of these amino acids are conserved between NiV_M_ and NiV_B_. | | | | | | | | | | | |
| SNP, single nucleotide polymorphism; SNP%, percentage of reads containing SNP, Ref Pos; Nucleotide position in reference genome; Depth, number of reads over base; Cnt, number counted. | | | | | | | | | | | |
| ^a^This is the NiV_M_ P2 isolate used for infections presented in this study.  ^b^The Reference strain used is GenBank Assession number AJ627196.1  ^c^This is a NiV_M_ P3 isolate used in previous m102.4 study^30^.  ^d^This is the NiV_B_ isolate used for infections presented in this study.  ^e^The Reference strain used is GenBank Assession number AY988601.1 | | | | | | | | | | | |

**Supplementary Table 2. NiV_B_ Serum Neutralization Titers^a^ in Control or m102.4 Treated** AGMs

| **Treatment Regimen** | **Subject No.** | **Day 0^b^** | | **Day 3** | **Day 5** | **Day 7** | **Day 15** | **Day 28** |
| --- | --- | --- | --- | --- | --- | --- | --- | --- |
|  |  |  | |  |  |  |  |  |
| Days 1 and 3 | O7698 | <20 | | 2560 | 5120 | 5120 | 2560 | 1280 |
|  | O8215 | <20 | | 2560 | 5120 | 5120 | 2560 | 1280 |
|  | O8214 | <20 | | 2560 | 2560 | 5120 | 2560 | 1280 |
|  |  |  | |  |  |  |  |  |
| Days 3 and 5 | O7522 | <20 | | 80 | 1280 | 5120 | 640 | 640 |
|  | O8149 | <20 | | 80 | 2560 | 5120 | 1280 | 1280 |
|  | O8211 | <20 | | 160 | 2560 | 5120 | 1280 | 640 |
|  |  |  | |  |  |  |  |  |
| Days 5 and 7 | B4825 | <20 | | NT | <20 | 320 |  |  |
|  | B6484 | <20 | | NT | 160 | 640 |  |  |
|  | C3882 | <20 | | NT | 160 | 320 |  |  |
|  |  |  | |  |  |  |  |  |
| None | O7923 | <20 | | NT | NT | <20 |  |  |
|  | C4373 | <20 | | NT | NT | <20 |  |  |
|  |  | |  |  |  |  |  |  |

^a^ reciprocal serum dilution at which 50% of virus was neutralized

^b^day post-NiV challenge

NT- not tested
